# Factors prompting PSA-testing of asymptomatic men in a country with no guidelines: a national survey of general practitioners

**DOI:** 10.1186/1471-2296-10-3

**Published:** 2009-01-12

**Authors:** Frances J Drummond, Anne-Elie Carsin, Linda Sharp, Harry Comber

**Affiliations:** 1 National Cancer Registry, Ireland, Building 6800, Airport Business Park, Kinsale Rd, Cork, Ireland

## Abstract

**Background:**

Increased use of prostate specific antigen (PSA) has been associated with increased prostate cancer incidence. Ireland is estimated to have one of the highest prostate cancer incidences in Europe and has no national guidelines for prostate cancer screening. GPs have a pivotal role in influencing PSA testing, therefore, our aim was to describe GP testing practices and to identify factors influencing these.

**Methods:**

A postal survey, including questions on clinical practice and experience, knowledge and demographics was distributed to all GPs (n = 3,683). The main outcomes were (i) PSA testing asymptomatic men and (ii) "inappropriate" PSA testing, defined as testing asymptomatic men aged < 50 or > 75 years. Factors associated with these outcomes were identified using logistic regression.

**Results:**

1,625 GPs responded (response rate corrected for eligibility = 53%). Most respondents (79%) would PSA test asymptomatic men. Of these, 34% and 51% would test asymptomatic men < 50 and > 75 years, respectively. In multivariate analyses, GPs were more likely to test asymptomatic men if they were ≥ 50 years, in practice ≥ 10 years, female or less knowledgeable about PSA efficacy. Male GPs who would have a PSA test themselves were > 8-times more likely to PSA test asymptomatic men than GPs who would not have a test. GPs who had an asymptomatic patient diagnosed with prostate cancer following PSA testing, were > 3-times more likely to test asymptomatic men. Practice-related factors positively associated with testing included: running 'well man' clinics, performing occupational health checks and performing other tests routinely with PSA. Factors positively associated with 'inappropriate' testing included; being male and willing to have a PSA test, having worked/trained in the UK and supporting annual PSA testing. 91% of respondents supported the development of national PSA testing guidelines.

**Conclusion:**

Our findings suggest that widespread PSA testing of asymptomatic men in primary care is primarily due to a combination of clinical experience, poor knowledge and the support of doctors for PSA testing, as evidenced by the willingness of male doctors to have a PSA test. There is an urgent need for education and support for GPs concerning prostate cancer screening, starting with the implementation of national guidelines.

## Background

Screening for prostate cancer remains an important and controversial public health issue. Prostate specific antigen (PSA) testing of asymptomatic men, *de facto *screening, is increasingly common in many developed countries [1,2]. This escalating use of PSA testing has driven the increases in prostate cancer incidence that began to be seen in the 1990s, and continues to be observed [3,4]. However, a recent Cochrane review confirmed that evidence on the efficacy of PSA testing as a prostate cancer screening tool is still lacking [5] and mortality data from two large randomised control trials is a number of years away [6,7]. Thus, the question posed by Ransohoff and colleagues in 2002 [8] remains pertinent – why is prostate cancer screening so common when the evidence is so uncertain?

The majority of PSA tests originate in primary care [9,10]. While other factors such as urologists practice [11], local guidelines [11], patient demand [12], a man's social network [13] and the media [11-13] influence the frequency of PSA testing, GPs have a pivotal role in determining levels and patterns of testing in the population. Improving understanding of the triggers for PSA testing of asymptomatic men is, therefore, of considerable importance, especially if there is to be any possibility of evidence-based practice in this area.

Several studies have examined influences on GPs' attitudes towards PSA testing or prostate cancer screening. Patient- (including age [14], smoking-status [14], marital status [12-15]) and physician-related factors (including age [14], gender [13-16], non-solo practices [14-17], positive attitude towards screening for prostate cancer [15], belief in the efficacy of prostate cancer treatment [18], agreement with guidelines and being in private practice [19]) have been identified. However, most have emerged from univariate analyses and it is unclear whether each acts independently. In addition, all of the studies were performed in countries where guidelines are in place regarding the use of PSA testing, although whether these influence PSA testing practices is uncertain [16,17,19,20]. It would, therefore, be valuable to investigate the multi-dimensional nature of GP-related triggers for PSA testing of asymptomatic men in a setting where there are no "barriers" influencing GPs' attitudes and practices.

In Ireland, which has a mixed public-private healthcare system there was, until recently, no national policy or guidelines on PSA testing or prostate cancer screening; in 2006 the National Cancer Forum recommended against the introduction of a population-based prostate cancer screening programme [21]. PSA testing is on the increase; during 1999 to 2004, the number of PSA tests performed rose by almost 4-fold [9]. This was greater than the increase observed in Northern Ireland [2], where the NHS Executive and the National Screening Committee guidelines recommend against screening asymptomatic men [22]. These trends mean that Ireland is predicted to have had the highest rate of prostate cancer incidence in Europe in 2006 [23]. It was against this background that we investigated factors associated with active PSA testing of asymptomatic men among GPs.

## Methods

### Questionnaire

A self-administered postal questionnaire (Additional file 1) was developed with the aim of assessing the beliefs, practices, knowledge, and information needs of GPs regarding prostate cancer and PSA testing. Questions were included on personal and practice characteristics, knowledge of prostate cancer risk factors, including increasing age [24], family history of prostate cancer [25], high dietary fat intake [26], African American ethnicity [27] and PSA test properties (e.g. positive predictive value (PPV) [28], PSA testing practice, and patient management or referral following abnormal PSA results. As regards PSA practice, GPs were asked 'Do you usually use PSA to test asymptomatic men for prostate cancer? and if so, in what age groups (30 to 80 years and older). GPs were also asked 'Would you consider having a PSA test done yourself in the future?' The questionnaire was pre-tested on 12 GPs for acceptability, ease of completion, and to determine whether any important issues had been omitted, and revised accordingly. The final instrument consisted of 37 closed questions, 11 patient management scenarios [29] and a box for written comments, over 12 A4 pages. A copy of the questionnaire is available from the authors by request.

### Settings, subjects and questionnaire administration

Thirty-six laboratories measure PSA in the Republic of Ireland [11]. There are 38 practicing urologists for a population of 2.1 million men of all ages.

As described elsewhere, we created a database of all GPs believed to be practicing in the Republic of Ireland [30]. Briefly, a comprehensive, computerised, list was compiled from the Irish Medical Directory 2005/2006 (IMD), telephone directories, health authority lists of GPs with contracts to provide services to the Health Service Authority and a list, maintained by the National Cancer Registry, Ireland (NCR) since its establishment in 1994, of GPs who had referred cancer patients. This compiled list was compared with the membership directory of the Irish College of General Practitioners (ICGP). Our final list included 3,683 GPs.

Three weeks before questionnaires were dispatched, a pre-contact letter was sent to each GP informing them about the study and that they would be receiving the questionnaire [30]. The questionnaire was then sent to all 3,683 GPs, together with a pre-paid return envelope. Questionnaires were assigned numerical codes to ensure confidentiality. Those who had not returned the questionnaire after four weeks were sent a reminder. If necessary, a second reminder was sent after a further four weeks. Each reminder contained another copy of the questionnaire and pre-paid envelope. No inducements were offered for questionnaire completion.

During the course of the study, 590 GPs were found to be ineligible (retired, died, moved and another address was unavailable, did not see male patients > 40 years, or included twice) and excluded. The analysis, therefore, relates to 3,093 GPs.

To ascertain whether there was a difference between responders and non-responders regarding their PSA practice and beliefs, an abbreviated 1-page survey consisting of 8 key questions selected from the main questionnaire was sent to 34% (500/1,468) randomly selected non-responders, with a pre-paid return envelope.

### Outcomes and statistical analysis

The primary outcome was whether, or not, GPs usually used PSA to test asymptomatic men for prostate cancer. To investigate factors associated with 'inappropriate' PSA testing [31] defined for this study as testing asymptomatic men outside the age groups recommended by most published guidelines, the secondary outcome related to whether or not GPs usually tested 'asymptomatic' < 50 or > 75 years.

The analysis was based on those who responded to the full questionnaire and was done in Stata V 8.2 (StataCorp, 2003). Chi-square tests were used to assess univariate associations between (i) responders and non-responders to the full questionnaire and (ii) characteristics of the participants and the primary and secondary outcomes. Multivariate logistic regression were used to compute odds ratios (OR) and 95% confidence intervals for factors associated with the primary and secondary outcomes. Responses to all questions were considered as potential explanatory variables. The models were adjusted for variables which were associated with questionnaire response and those GP characteristics (gender; age; year of graduation; having qualifications in geriatric medicine, palliative care and occupational health; whether worked or trained in the UK; having a specialist interest in men's health, research, urological problems or cancer detection) and practice characteristics (being a fulltime principal; solo verses multi-doctor practice; percentage of patients having private health insurance; percentage of time performing occupational health assessments; involvement in GP training; running 'well man' clinics and having a policy on PSA testing) for which the likelihood ratio test p-value was < 0.1 were included in the models. Several factors were reduced to dichotomous variables for the purposes of analysis; age of GP (< 50, > 50 years), the age at which PSA testing usually starts; whether (1% to > 75%) or not (0%) they performed occupational health checks; preferred frequency of PSA testing (annual or more frequent verses less frequent testing (biennially, more than biennially, when a man develops symptoms)); whether or not they refer men to a urologist at a PSA level ≥ 7 ng/ml (only 5% of respondents reported that they would refer a 55 year old man to a urologist at a PSA level of 2.5–3.9 ng/ml, 3% would refer a 65 year old man at this PSA level); whether they did, or did not, overestimate the PPV of PSA alone, and PSA with digital rectal exam (DRE), defined as 25% and 48%, respectively, in a meta-analysis [28]. A score based on the seven questions related to prostate cancer risk factors was built by summing up the number of correct answers. Interactions between variables were investigated: none were sufficiently important or meaningful in statistical terms or as regards interpretation to justify incorporation into the final models. Because of the inter-relationships between factors in the questionnaire (particularly GPs' personal and practice characteristics), we took particular care to avoid multicollinearity in the model fitting. We computed variance inflation factors and tolerances for the variables in the final models; all had low variance inflation factors and high tolerance suggesting collinearity was not a problem. Model fit was assessed using the Hosmer and Lemeshow's test and the final models had adequate fit.

We assumed *a priori *that the questionnaire response rate would be 50%. Assuming that 65% of respondents would be willing, and 35% unwilling, to test asymptomatic men [16], and adjusting for a variance inflation factor of 1.4 [32], the study would have 88% power to detect as statistically significant a multivariate odds ratio of 1.5 associated with a risk/explanatory factor prevalent in one-third of the GPs unwilling to test asymptomatic men (α = 0.05, two-sided test).

## Results

### Response rate and participants' characteristics

The response rate for the full questionnaire was 53% (1,625/3,093). Responders and non-responders did not differ by gender (p = 0.711), but did differ by year of graduation and practice area; GPs who graduated before 1970 or worked in the Eastern Regional Health Authority, which includes the capital city, Dublin, were significantly less likely to participate (p < 0.001). All multivariate analyses were, therefore, adjusted for age and health board area.

Characteristics of the responding GPs are presented in Table 1. The demographic and practice characteristics of respondents did not differ from that described by O'Dowd et al (2005) [33].

**Table 1:** Characteristics of responding GPs

Demographic characteristic		Number (%)
Gender	Male	1094 (67%)
	Female	529 (33%)
Full time principals	Yes	1287 (79%)
Age (years)	≤ 39	305 (19%)
	40–49	516 (32%)
	50–59	534 (33%)
	> 60	253 (16%)
Time in general practice (years)	≤ 5	139 (9%)
	6–10	196 (12%)
	11–20	488 (30%)
	21–30	480 (30%)
	> 30	293 (18%)
No. sessions as a GP Per week	≤ 5	156 (10%)
	6–8	391 (24%)
	9–11	875 (54%)
	> 11	150 (9%)
No. GPs per practice	1	575 (35%)
	> 1	990 (61%)
	Missing	59 (4%)
Area of practice	ERHA	478 (29%)
MICGP	Yes	1063 (65%)

MICGP: Member of the Irish College of General Practitioners

ERHA: Eastern Regional Health Authority

Almost half of responders to the survey had worked or trained in another country (49%), the majority of these in the UK (72%). Just over one-third of respondents (34%) were actively involved in teaching and 70% undertook occupational health assessments. Fourteen percent of responding GPs had held a postgraduate post in urology. Of the respondents, 14% reported that they had a specialised interest in mens' health, while 7% and 3% had specialist interests in cancer detection and urological problems, respectively. The practices of 17% of GPs held a "well man" clinic or something similar. Sixty percent of GPs attended an educational meeting where PSA was a main topic, 27% of these within the year prior to the survey.

### Knowledge of prostate cancer risk factors and PSA test properties

Just over one-third (35%) of respondents correctly answered 4 or more of the 7 questions on prostate cancer risk factors, 2% correctly answered all seven questions. The majority of respondents correctly identified increasing age as a prostate cancer risk factor, while 20% and 76%, respectively, were unaware that having a first degree relative with prostate cancer and having a high dietary fat intake increases a man's risk of developing the disease (Table 2). Over half (56%) responded that smoking was a risk factor and 28% incorrectly described benign prostate hyperplasia as increasing prostate cancer risk. Only 17% of respondents recognised that African American ethnicity was associated with a higher risk of prostate cancer.

**Table 2:** Knowledge of prostate cancer risk factors; we asked GPs 'For each of the following, please indicate whether you believe they influence the risk of developing prostate cancer.

**Risk factor**	**Correct** **N (%)**	**Does not effect risk** **N (%)**	**Reduces risk** **N (%)**	**Increases risk** **N (%)**	**Don't know** **N (%)**	**Missing** **N (%)**
Increased age (> 50 years)	1,592 (98%)	8 (0.5%)	3 (0.2%)	1,592 (98%)	4 (0.2%)	18 (1%)

1^st ^degree relative with prostate cancer	1,300 (80%)	97 (6%)	3 (0.2%)	1,300 (80%)	184 (11%)	41 (3%)

Current smoking	263 (16%)	263 (16%)	3 (0.2%)	908 (56%)	386 (24%)	65 (4%)

High dietary fat intake	386 (24%)	391 (24%)	8 (0.5%)	386 (24%)	777 (48%)	63 (4%)

1^st ^degree relative with breast cancer	298 (18%)	533 (33%)	2 (0.1%)	298 (18%)	725 (45%)	67 (4%)

Benign Prostatic Hyperplasia	857 (53%)	857 (53%)	13 (0.8%)	452 (28%)	247 (15%)	56 (3%)

African American ethnicity	284 (17%)	109 (7%)	104 (6%)	284 (17%)	1,078 (66%)	50 (3%)

More than half of GPs (54%) overestimated the likelihood that a positive PSA result indicated prostate cancer (PPV > 30% [28]) and 68% overestimated the PPV of PSA and DRE (PPV > 50% [28]) combined (Table 3).

**Table 3:** Knowledge of the positive predictive value of PSA and Digital Rectal Exam (DRE); we asked 'For the following tests, what is the likelihood that a positive result indicates prostate cancer (positive predictive value)?'

**Test**	**< 10%** **N (%)**	**10–30%** **N (%)**	**30–50%** **N (%)**	**> 50%** **N (%)**	**Not sure** **N (%)**	**Missing** **N (%)**
PSA level	107 (7%)	464 (29%)	491 (30%)	396 (24%)	91 (6%)	76 (5%)

DRE and PSA	6 (0.4%)	67 (4%)	277 (17%)	1,110 (68%)	96 (6%)	69 (4%)

### PSA testing practice

More than three-quarters of respondents (79%) reported that they would usually use PSA to test asymptomatic men for prostate cancer. Two thirds of these GPs (65%) would test asymptomatic men outside the recommended age-ranges; 34% and 51% would test men younger than 50 years and older than 75 years, respectively. Of the respondents, 69% would frequently PSA test a man attending with lower urinary tract symptoms, 80% would frequently PSA test a man with a family history of prostate cancer, 11% would frequently test men with a family history of breast cancer and 32% would PSA test a man as part of an occupational health assessment. Almost one-third of GPs frequently actively arrange appointments for PSA testing (29%) and 55% perform other blood tests routinely with PSA.

Most GPs (90%) reported that they frequently inform the patient that his PSA is being checked. Only 5% frequently ask about ejaculation in the week preceding a PSA test, 28% sometimes and 14% rarely or never discuss the implications of an abnormal test, prior to conducting the test. Twelve percent frequently discuss prostate cancer treatment, prior to testing.

The majority of male GPs (88%), both younger (84%) and older (91%) than 50 years, reported that they would have a PSA test themselves.

Forty percent of all respondents believe men should be PSA tested biennially, while 30% support annual or more frequent testing. GPs who believe that testing men less frequently than biennially (11%) or only when symptoms develop (16%) were in the minority.

### Information needs

More than two-thirds of GPs (69%) wanted information on PSA testing. Overall 82% requested information on at least one of the topics listed, 36% wanted information on all topics (Table 4). The overwhelming majority of respondents were in favour of national guidelines on prostate cancer screening (92%) and PSA testing in general practice (91%).

**Table 4:** Information needs; we asked GPs 'Do you feel you need more information with regard to:'

**Topics**	**Yes** **N (%)**
PSA Testing	1,116 (69%)

Prostate cancer risk factors	1,164 (72%)

Prostate cancer diagnosis/detection	1,034 (64%)

Prostate cancer treatment	931 (57%)

Prostate cancer survival	823 (51%)

All of the above	589 (36%)

### Factors associated with the PSA testing behaviour of GPs

In the multivariate model, female GPs, full-time principals, those older than 50 years, in practice more than ten years, or who had a specialist interest in cancer detection, were significantly more likely to usually test asymptomatic men (p < 0.05) (Table 5). GPs who undertook occupational health assessments and whose practice offered well-man clinics were more than 50% more likely to usually test asymptomatic men.

**Table 5:** Factors significantly associated with the propensity of GPs to testing asymptomatic men

		**Yes**		**Total**	**Univariate**	**Multivariate**
		**Num**	***%***	**Num**	**OR [95%CI]**	**OR [95%CI]**
***GP Characteristics***						
**Gender**	Male	867	*79.7*	1,088	1.00	1.00
	Female	408	*79.1*	516	0.96 [0.74,1.25]	**1.49 [1.08,2.05]**
						p = 0.016
**Age group**	< 50 years	604	*74.3*	813	1.00	1.00
	≥ 50 years	660	*85.1*	776	**1.97 [1.53,2.54]**	**1.71 [1.27,2.31]**
						p = 0.002
**Full-time principal**	Yes	1,038	*81.4*	1,275	1.00	1.00
	No	225	*71.9*	313	**0.58 [0.44,0.78]**	**0.57 [0.41,0.81]**
						p = 0.006
**Length of Practice**^1^	≤ 10 years	235	*70.6*	333	1	1
	11–20 years	366	*75.9*	482	1.32 [0.96,1.80]	**1.45 [1.02,2.07]**
	21–30 years	406	*85.5*	475	**2.45 [1.73,3.47]**	**2.39 [1.60,3.56]**
	≥ 30 years	248	*86.1*	288	**2.59 [1.72,3.89]**	**2.51 [1.57,4.03]**
						p < 0.001
						
***Special interest in:***						
**Research**	No	1,251	80.2	1,559	1.00	1.00
	Yes	24	53.3	45	**0.28 [0.15,0.51]**	**0.39 [0.20,0.77]**
						p = 0.008
**Cancer detection**	No	1,166	78.3	1,489	1.00	1.00
	Yes	109	94.8	115	**5.03 [2.19,11.55]**	**3.61 [1.48,8.85]**
						p = 0.001
						
***Practice characteristics***						
**Occupational Health Assessments**	No	342	*72.6*	471	1.00	1.00
	Yes	907	*82.6*	1,068	**1.79 [1.39,2.31]**	**1.68 [1.27,2.22]**
						p = 0.001
**Actively involved in GP teaching**	No	865	*82.5*	1,049	1.00	1.00
	Yes	392	*73.4*	534	**0.59 [0.46,0.75]**	**0.61 [0.46,0.80]**
						p = 0.002
**Run'Well man' clinic/appointments**	No	541	*75.2*	1,311	1.00	1.00
	Yes	721	*83.4*	278	**1.91 [1.31,2.77]**	**1.59 [1.06,2.39]**
						p = 0.064
**Have a policy on PSA testing**	No	865	*75.2*	719	1.00	1.00
	Yes	392	*83.4*	865	**1.67 [1.31,2.13]**	**1.45 [1.11,1.90]**
						p = 0.007
						
***Knowledge***						
**PPV PSA level**	< 30%	472	*71.6*	659	1.00	1.00
	Overestimated (> 30%)	743	*84.5*	879	**2.16 [1.68,2.78]**	**2.02 [1.55,2.63]**
						p < 0.001
**PPV PSA&DRE**	< 50%	310	*69.8*	444	1.00	1.00
	Overestimated (> 50%)	912	*83.0*	1,099	**2.11 [1.63,2.72]**	**2.06 [1.56,2.73]**
						p < 0.001
						
***Clinical Practice***						
**Refer 55 yr man at PSA level**	≤ 7 ng/ml	803	*82.9*	969	1.00	1.00
	≥ 7 ng/ml	332	*74.9*	443	**0.62 [0.47,0.81]**	**0.60 [0.45,0.81]**
	Lab reference range	129	*73.7*	175	**0.58 [0.40,0.84]**	**0.61 [0.40,0.93]**
						p = 0.002
**Frequency of PSA testing: Annually or less**	Yes	457	*94.2*	485	**6.01 [4.01,9.01]**	**5.54 [3.64,8.45]**
	No	793	*73.1*	1,085	1.00	1.00
						p < 0.001
**Perform other routine tests with PSA**	No	764	*85.6*	893	1.00	1.00
	Yes	505	*71.6*	705	**2.35 [1.83,3.01]**	**1.97 [1.51,2.57]**
						p < 0.001
**Inform patient prior to testing**	Frequently	1,193	*81.9*	1,457	1.00	1.00
	Sometimes	69	*68.3*	101	**0.48 [0.31,0.74]**	**0.53 [0.31,0.89]**
	Rarely/Never	9	*27.3*	33	**0.08 [0.04,0.18]**	**0.09 [0.04,0.23]**
						p < 0.001
**Had an asymptomatic man diagnosed with PCa via**						
**PSA testing**	No	338	*64.4*	525	1.00	1.00
	Yes	923	*87.0*	1,061	**3.70 [2.87,4.76]**	**3.31 [2.51,4.37]**
						p < 0.001
**Practice changed post PSA-detected PCa**^2^	No	265	*78.6*	337	1.00	1.00
	Yes	558	*91.5*	610	**2.92 [1.98,4.29]**	**2.78 [1.81,4.25]**
						p < 0.001
**Consider having a PSA test yourself**^3^	No	48	*37.8*	127	1.00	1.00
	Yes	807	*85.7*	942	**9.80 [6.56,14.65]**	**8.25 [5.26,12.93]**
						p < 0.001

The multivariate model was fully adjusted for characteristics of the GP (age, gender, full-time principal, health board, whether they had qualifications in geriatric medicine, palliative care or occupational health, whether they trained in UK, whether they had a specialist interest in men's health, research, urological problems or cancer detection) and the practice (solo vs multi-doctor practice, percent of patients with private health insurance, percent of time performing Occupational Health Assessments, involvement in GP teaching, running a "well man" clinic, and having a practice policy on PSA testing).

^1^Length of time in practice was not adjusted for age because they were correlated.

^2^Among GPs who had an asymptomatic man diagnosed with prostate cancer via PSA testing (n = 1,061)

^3^Among male GPs (n = 1,088)

PPV = Positive Predictive Value.

PCa = Prostate Cancer

DRE = Digital Rectal Exam

GPs that had an asymptomatic patient diagnosed with prostate cancer through a PSA test were 3.31-times more likely to test asymptomatic men than those who had not. In further analysis we found that this effect was particularly pronounced among older GPs; compared to GPs aged < 50 who had not has a patient diagnosed with prostate cancer through a PSA test, GPs aged ≥ 50 who had had such a patient were 6-times more likely to routinely PSA test asymptomatic men (multivariate OR = 6.11, 95% CI 3.96–9.25).

Male GPs who would have a PSA test were 8.25-times more likely to test asymptomatic men than GPs who would not have a test; this was the highest OR from the multivariate model.

GPs who were actively involved in teaching were 39% less likely to usually test asymptomatic men and the likelihood of testing was reduced by 61% among GPs with an active interest in research. Doctors who overestimated the PPV of PSA and/or DRE, those who favoured annual or more frequent testing for men 50 years and older, and those with a personal or practice PSA testing policy had a greater likelihood of testing asymptomatic men. Respondents with a greater knowledge of prostate cancer risk factors were less likely to usually test asymptomatic men in univariate analyses, however, this association was not significant following adjustment for other factors. There was no relationship between the desire for more information about PSA and testing behaviour, in either univariate or multivariate analyses.

The percentage of patients with medical cards (which are means-tested and entitle the bearer to receive certain health services, including GP visits, free of charge) or with private health insurance was not associated with PSA testing practice in the multivariate model. Whether the GP had trained in the UK, worked in a solo or multi-doctor practice, had attended an education meeting on PSA, or had an interest in men's health was unrelated to the likelihood of testing.

GPs who test asymptomatic men had a very favourable attitude towards regular testing – they were 5.54-times more likely to believe that men should have annual or more frequent PSA testing than GPs who would not test asymptomatic men.

There was no significant association observed between those GPs who believed that aggressive treatment (prostatectomy and/or radiotherapy) or those who believed that non-aggressive treatment (watchful waiting and/or hormone therapy) was more appropriate for the treatment of prostate cancer, and the likelihood of PSA testing asymptomatic men (OR 0.93 95%CI = 0.68,1.28).

### Factors associated with favouring PSA testing younger or older asymptomatic men

In the secondary analysis, GPs who trained or worked in the UK, had a positive attitude towards regular testing (annual or less), who perform other tests routinely with PSA, who had a policy on PSA testing and who would refer a young man (55 years) to a urologist at a lower PSA level (≤ 7 ng/ml) were all independent factors associated with the inappropriate use of PSA (Table 6). Male GPs who would have a PSA test were twice as likely to PSA test asymptomatic men younger than 50 years and older than 75 years. GPs with an MD or an occupational health qualification were less likely to inappropriately use PSA. Having an asymptomatic patient diagnosed with prostate cancer detected via PSA testing was not significantly associated with PSA testing in these age groups, in the multivariate model. The models were re-run to investigate, separately, the factors influencing whether GPs would routinely test asymptomatic men younger than 50 years and 75 and older, and the results did not differ to any great extent (data not shown).

**Table 6:** Factors associated with PSA testing asymptomatic men < 50 and > 75 years.

		**yes**		**Total**	**Univariate**	**Multivariate**
		**Num**	***%***	**Num**	**OR [95%CI]**	**OR [95%CI]**
***GP Characteristics***						
**Gender**	Male	564	*65.1*	867	1.00	1.00
	Female	268	*65.7*	408	1.03 [0.80,1.32]	1.07 [0.82,1.41]
						P = 0.615
**Age group**	< 50 years	394	*65.2*	604	1.00	1.00
	≥ 50 years	429	*65.0*	660	0.99 [0.79,1.25]	0.97 [0.75,1.26]
						P = 0.513
**Worked or trained in UK**	No	519	*63.5*	817	1.00	1.00
	Yes	313	*68.3*	458	1.23 [0.97,1.58]	**1.35 [1.05,1.74]**
						P = 0.020
**Post-graduate qualifications**						
**Occupational Health**	No	731	66.3	1,103	1.00	1.00
	Yes	61	53.5	114	**0.59 [0.40,0.86]**	0.67 [0.45,1.00]
						P = 0.051
**MD**	No	824	65.8	1,252	1.00	1.00
	Yes	8	34.8	23	**0.28 [0.12,0.66]**	**0.30 [0.12,0.73]**
						P = 0.008
						
***Clinical Practice***						
**Lowest PSA level at which to refer a 55 yr old man for urological assessment**					1.00	1.00
	≤ 7 ng/ml	541	*67.4*	803		
	≥ 7 ng/ml	206	*62.0*	332	0.79 [0.61,1.03]	0.72 **[0.54,0.95]**
	Lab reference range	77	*59.7*	129	0.72 [0.49,1.05]	0.74 [0.59,1.11]
						P = 0.062
**How often should a PSA test be performed in men aged ≥ 50 years?**						
**Annually or less?**	No	472	*59.5*	793	1.00	1.00
	Yes	347	*75.9*	457	**2.15 [1.66,2.77]**	**2.08 [1.59,2.71]**
					p < 0.001	p < 0.001
**Do you have a policy on PSA testing**						
	No	345	*62.3*	554	1.00	1.00
	Yes	487	*67.5*	721	1.26 [1.00,1.59]	**1.36 [1.06,1.75]**
						p = 0.014
**Do you perform other tests routinely with PSA**						
	Yes	532	*69.6*	764	1.00	1.00
	No	299	*59.2*	505	**0.63 [0.50,0.80]**	**0.63 [0.49,0.81]**
						p < 0.001
**Asymptomatic man diagnosed with PCa via PSA testing**						
	Yes	617	*66.8*	923	1.27 [0.99,1.65]	1.24 [0.94,1.63]
	No	207	*61.2*	338	1.00	1.00
						p = 0.205
**Consider having a PSA test yourself**						
	No	23	*47.9*	48	1.00	1.00
	Yes	541	*66.1*	819	**2.15 [1.20,3.86]**	**2.02 [1.10,3.72]**
						p = 0.034

The multivariate model was fully adjusted for age, gender, health board, having more than 50% patients with private health insurance, having worked or trained in UK, having a policy regarding PSA testing and having an occupational health or an MD post-graduate qualification. PCa = prostate cancer

### Non-response bias

The brief 1-page survey was returned by 175 of the sample of 500 non-responders to whom it was sent (35%), taking the combined response rate to the full questionnaire or the abbreviated survey to 58%. These 175 GPs were significantly more likely to i) be older (> 50 years, p = 0.023), ii) test asymptomatic men (p = 0.007) iii) PSA test men younger than 40 years (p = 0.008), and iv) to PSA test men older than 75 years (p = 0.034) and v) were also more likely to have a PSA test themselves (p = 0.112) than responders to the initial survey instrument.

## Discussion

In the absence of national guidelines, this survey shows that the routine practice of most GPs working in Ireland is to test asymptomatic men for prostate cancer by PSA testing. PSA testing [1,2], along with other factors including biopsy and TURP practices [34], have lead to an increase in the detection of prostate cancer [3,4]. Given that the international consensus is either negative or neutral on the value of prostate screening [35], the undoubted financial [36], physical [37] and mental [38] costs, and the fact that Ireland is predicted to have had the highest prostate cancer incidence in Europe in 2006 [23], it is important to identify the determinants of this testing behaviour and to enquire as to methods which might address it.

A dominant theme emerging from this survey is the approach of GPs to clinical decision-making. Our results indicate that younger GPs, those involved in education and research and those with more information on PSA and prostate cancer were less likely to test asymptomatic men, although, interestingly, they were not more discriminating with regard to the age at testing. Older GPs, women (whose routine exposure to prostate cancer is likely to be infrequent), those with less education and research exposure seemed to be more likely to perhaps trust their clinical experience, however limited. We suggest that these beliefs were sincerely held, and unlikely to be due to either patient demand or method of reimbursement, as evidenced by the GPs' own willingness to undergo PSA testing and also the lack of difference in testing practices observed between private patients (reimbursed on a fee-per-item basis) and public patients (capitation).

How has it come about that the majority of Irish GPs hold beliefs which seem in conflict with the scientific evidence? Many of the GPs responding to this survey were not well informed about prostate cancer or PSA efficacy. Unavailability of information seems an implausible explanation; the internet has placed a vast volume of information, original papers, reviews and guidelines at the fingertips of even the most isolated GP. In addition, our study has shown that, in contrast to the findings in Northern Ireland [16], attendance at educational meetings on PSA had in itself a negligible effect on practice, and furthermore level of testing activity was not correlated with the GPs perceived need for more information. However, information on PSA testing and opinion of expert groups is often conflicting [35] and PSA has in general received a positive press, with the various media campaigns [39] not dwelling on the limitations of PSA as a tumour marker. Following distribution of this survey, the National Cancer Forum published 'A Strategy for Cancer Control in Ireland' (2006) in which they recommended against the use of PSA for population-based prostate cancer screening [40]. However, preliminary evidence suggests that this policy has not affected the level of PSA testing in Ireland [11]. Improvements in the methods of dissemination of information [41,42] and evaluation of the effectiveness of the information may be necessary if public health policies are going to impact on general practice.

A propensity to be influenced by a single incident, a diagnosis of prostate cancer by PSA testing, seemed to be central to the belief system of the high screening GPs. Our findings suggest that the GPs may be less inclined to trust or apply "evidence-based" practice, feeling that it cannot do justice to the heterogeneity of patients and pathology in general practice, and trust more to their salient experience and "gut feelings". This, in the case of PSA testing, seems a reasonable strategy. Most GPs will see on average no more than one case of prostate cancer every 1.5 years [43] and so the early diagnosis of a case can easily seem to be a "success" for PSA testing, given the emphasis placed on the early diagnosis of cancer. There are few, if any, negative implications for a GP in carrying out a test for diagnosing a cancer [8], while "missing" a cancer would be seen as negligent [44,45] and would be a cause of decisional regret for the doctor [46,47]. Fear of litigation in such a case may also be a potent factor [48]. It is difficult, in these circumstances, for a GP to put the "common good" ahead of what seem to be the best interest of the patient. In support of this, GPs perceive that the implementation of national guidelines in this area would afford them some protection from litigation [12].

GPs who worked in the UK were more likely to test asymptomatic men outside of the recommended age groups. The reasons for this are unclear. Despite the fact that guidelines do not recommend prostate cancer screening in the UK [22], awareness of these guidelines among GPs was low [16,20]. Two studies have shown that they did not influence the PSA testing or referral practices of GPs [16,20] and there is evidence of prostate cancer screening in the UK [2,34,49-51]. A survey of GPs in the UK revealed that, similar to the findings of this study, 76% of GPs would PSA test asymptomatic men [52]. One possibility is that GPs who worked or trained in the UK were exposed to PSA in a clinical setting before those who did not work outside of Ireland; the rate PSA testing peaked in the mid-1990's in Northern Ireland [2] and Scotland [34], while the increase in PSA testing in Ireland did not start until after this time [9] and more than half of the laboratories in Ireland did not begin measuring PSA until post-1995 [11].

### Strengths and weaknesses

A major strength of this study is the large sample size and the national coverage obtained. We have built a comprehensive multivariate model of a cohort of GPs only and identified independent significant factors associated with PSA testing, as well as calculating the magnitude of these factors on PSA testing behaviour.

This survey was largely quantitative and could not explore GPs' beliefs and motivations in depth. However, given this limitation, some clear conclusions emerge. The main potential limitation of the study was that the analysis was based on GPs' self-reported PSA testing/screening behaviour. Our survey is therefore, subject to social bias whereby GPs may perceive the correct answer to be that they screen asymptomatic patients and themselves for prostate cancer. However, the number of PSA tests has increased 4-fold between 1999 and 2004 in Ireland and the majority of requests originate in general practice [9]. Use of surveys raises the possibility of respondent bias. When non-responders were analysed, they were significantly more likely to test asymptomatic men and to test asymptomatic men outside the appropriate age groups, than responders to the initial survey. Therefore, the PSA testing behaviour of responders may be more conservative than that of non-responders.

There are numerous mediolegal pitfalls in the diagnosis of prostate cancer [45], and indeed these were raised by a number of respondents [12], however, we did not investigate this factor as a potential predictor of GP testing behaviour. Further research is required into the influence of mediocolegal factors on PSA testing behaviour.

We found that attending educational meetings where PSA testing was a major topic had no effect on the PSA testing practice of GPs responding to this survey. However, we do not have any information about the structure and content of these meetings or indeed about who organized and sponsored them – all of which could potentially influence their effectiveness.

GPs who reported that they had a policy on PSA testing were significantly more likely to report that they would PSA test asymptomatic men, both within and outside the appropriate age groups. However, we do not know the details of the policies, the reasons for implementing them or the evidence used to inform them. This requires further research and is potentially an area of GP practice which could be targeted to effect change in PSA testing practice.

### Recommendations

Highlighted in this study is the need for further education and support for GPs concerning prostate cancer screening and the disconnection between the scientific literature on the value of PSA and clinical practice. Given the increase in patient knowledge, emphasis on evidence-based medicine and shared decision-making, there is a need for both doctor and patient to be fully informed [53]. This raises important questions and challenges regarding knowledge translation. The first step in closing the evidence-to-practice gap [41,42,54] usually involves developing evidence-based policy or guidelines. While the majority of GPs in our study agreed that there was a need for national guidelines on PSA testing in Ireland, as discussed it is unlikely that these alone would influence practice [16,20]. The lack of success of guidelines in changing the practice of health care providers may result from the passive dissemination of guidelines and policy [55] and more active approaches such as audit and feedback, reminders and educational outreach [56] are needed. In parallel, a programme of public education, along with methods to evaluate their effectiveness, and research into how men in Ireland are making decisions about prostate cancer screening is warranted, given the impact men's beliefs have on their decision to have a PSA test [57].

## Conclusion

Addressing this gap between evidence and practice needs a closer study of how GPs handle evidence; are GPs' poorly informed because of lack of opportunity, poor dissemination strategies or, having had a significant incident, do they make their minds up and cease to register any conflicting evidence? Do some GPs drift into testing, while others research the evidence before beginning? Our knowledge of the factors which bridge the gap between evidence and GPs beliefs, attitudes and practice remains rudimentary. Overall the belief of GPs in the clinical utility of PSA testing asymptomatic men, despite the lack of evidence that it improves outcome, has major implications for policy makers. If there is to be any hope of more evidence-based practice in this area, there is an urgent need to identify specific barriers to evidence-based decision making and to develop effective interventions and strategies to influence clinical practice and/or encourage adherence to evidence-based guidelines and policy.

## Competing interests

The authors declare that they have no competing interests.

## Authors' contributions

FD was the study coordinator, participated in study design, questionnaire design, conduct of the study, analysis of the study, writing up and revisions of drafts of the manuscript. LS participated in study design, questionnaire design, analysis of the study and revisions of drafts of the manuscript. A-EC was involved in data analysis and revision of drafts of the manuscript. HC was principle investigator, participated in protocol development, study design and revisions of drafts of the manuscript. All authors read and approved the final manuscript.

## Pre-publication history

The pre-publication history for this paper can be accessed here:



## Supplementary Material

Additional file 1 **PSA questionnaire**. A copy of the postal survey given to all GPs. Click here for file
