# Supplementary material for: Factors prompting PSA-testing of asymptomatic men in a country with no guidelines: a national survey of general practitioners
Source: BMC Fam Pract. 2009 Jan 12;10:3. doi: 10.1186/1471-2296-10-3 (PMC2646704; doi:10.1186/1471-2296-10-3)
Supplement: Additional file 1 — PSA questionnaire. A copy of the postal survey given to all GPs. [file 1471-2296-10-3-S1.doc]

**Prostate Specific Antigen**

**Survey**

*Thank you for taking the time to complete this questionnaire*


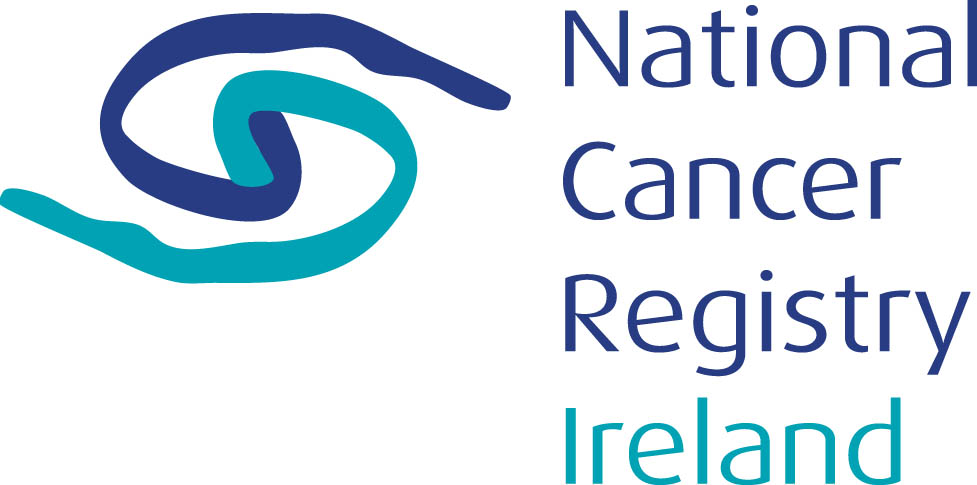


**ID**

The ***aim*** of this survey is to assess the attitudes and practices of Irish practitioners to Prostate Specific Antigen (PSA) testing.

The questionnaire will take approximately ***15 minutes*** to complete. Please fill in the appropriate tick boxes or write your answer in the spaces provided. Your answers will be kept strictly ***confidential***. Please return the completed questionnaire to the NCR in the pre-paid envelope provided.

If you have any ***questions***, please do not hesitate to contact the study coordinator:

Dr. Frances Drummond, Phd

Study coordinator,

National cancer Registry,

Elm Court, Boreenmanna road

Cork.

Tel: (021) 4703931; Fax: 021 4318016; email: [f.drummond@ncri.ie](mailto:f.drummond@ncri.ie); www.ncri.ie

**SECTION A: GP DETAILS**

**G1. How would you classify yourself?**

Full time principal 1 Part-time principal 2 Trainee 3

Locum 4 Not practicing 5 Other 6

If Other, please describe:

**G2. How many sessions do you work per week as a GP?**

0 1 1-2 2 3-5 3 6-8 4 9-11 5 >11 6

**G3. What sex are you?**  Male 1 Female 2

**G4. What age are you?** <30 1 31–39 2 40–49 3 50–59 4 >60 5

**G5. How long have you been in general practice (years)?**

<1 1 1-5 2 6-10 3 11-20 4 21-30 5 >30 6

**G6. Do you have any of the following postgraduate qualifications?**

| MICGP |  1 | MICGP |  6 |
| --- | --- | --- | --- |
| Diploma in Obstetrics |  2 | Diploma in Obstetrics |  7 |
| Diploma in Geriatric Medicine |  3 | Diploma in Geriatric Medicine |  8 |
| MRCPI/FRCSI |  4 | MRCPI/FRCSI |  9 |
| MD |  5 | MD | 10 |

If Other, please describe:

**G7. Did you work or complete any of your training in another country**? Yes 1 No 2

If Yes, where was it? U.K 1 USA 2 Other 3

If Other, please state:

**G8. Do you have a special area of interest?** Yes 1 No 2

If Yes, what is your area of interest?

Men’s health 1 Palliative care 2 Research 3

Urological problems 4 Cancer detection/screening 5 Other 6

If Other, please state:

**G9. Have you ever held a postgraduate post in urology?** Yes 1 No 2

If Yes:

Urological problems 4 Cancer detection/screening 5 Other 6

If Yes,

A. At what level was it? Intern 1 SHO 2 Register 3 Other 4 4

If Other, please state:

B. How long was the post for? <1 month 1 1-6 months 2 7-12 months 3 Other 4

If Other, please describe:

**G10. Have you attended postgraduate education meetings where the main topic was Prostate Specific Antigen (PSA) testing?**

Yes 1 No 2

If Yes, when was the most recent? Within the last year 1 1- 5 yrs ago 2 >5 yrs ago 3

**SECTION B: PRACTICE DETAILS**

**P1. How many doctors are in your practice?**  How many full-time equivalents?

How many full time GPs?

How many part-time GPs

Comments:

**P2. What is the total population of your practice? (Not just your personal list)**

(If you don’t know exactly, please give your best estimate.)

**P3. What percentage of your patients hold the following?** (If you don’t know exactly, please give your best estimate)

|  | **0%** | **1-24%** | **25-49%** | **50-74%** | **>75%** |
| --- | --- | --- | --- | --- | --- |
| A. A medical card |  |  |  |  |  |
| B. Private health insurance |  |  |  |  |  |

**P4. What percentage of your time is spent seeing patients for Occupational Health Assessments?**

0% 1 1-25% 2 26-50% 3 51-75% 4 >75% 5

**P5: Are you actively involved in General Practice teaching?** Yes 1 No 2

Medical students 1 GP Trainees 2 Other 3

If Other, please describe:

**P6. Does your practice run a ‘Well Man’ Clinic or something similar?** Yes 1 No 2

If Yes:

Urological problems 4 Cancer detection/screening 5 Other 6

If Yes,

A. Are these clinics held? Weekly 1 Fortnightly 2 Monthly 3 Bimonthly 4 Other 5 4

If Other, please state:

B. How many men attend each clinic? <10 1 11-20 2 21-30 3 >30 4 Other 5

If Other, please describe:

**SECTION C: PROSTATE CANCER TESTING PRACTICE**

C1. For each of the following, please indicate whether you believe they influence the risk of developing prostate cancer:

|  | **Does not affect risk** | **Reduces risk** | **Increases risk** | **Don’t know** |
| --- | --- | --- | --- | --- |
| A. Increased age (over 50 years) | 1 | 2 | 3 | 4 |
| B. 1^st^ degree relative with prostate cancer | 1 | 2 | 3 | 4 |
| C. Current smoking | 1 | 2 | 3 | 4 |
| D. High dietary fat intake | 1 | 2 | 3 | 4 |
| E. 1^st^ degree relative with breast cancer | 1 | 2 | 3 | 4 |
| F. Benign Prostatic Hyperplasia | 1 | 2 | 3 | 4 |
| G. African American ethnicity | 1 | 2 | 3 | 4 |

C2. For the following tests, what is the likelihood that a positive result indicates prostate cancer (positive predictive value)? (Prostate specific antigen (PSA); digital Rectal Exam (DRE); Transrectal ultrasound (TRUS)):

|  | **<10%** | **10-30%** | **30-50%** | **>50%** | **Not sure** |
| --- | --- | --- | --- | --- | --- |
| A. PSA level | 1 | 2 | 3 | 4 | 4 |
| B. DRE | 1 | 2 | 3 | 4 | 4 |
| C. PSA and DRE | 1 | 2 | 3 | 4 | 4 |

**C3. These questions relate to when, and on whom, you perform PSA testing. Do you:**

|  | **Frequently** | **Sometimes** | **Rarely** | **Never** |
| --- | --- | --- | --- | --- |
| A. actively arrange appointments for PSA testing? | 1 | 2 | 3 | 4 |
| B. PSA test men who attend with unrelated complaints? | 1 | 2 | 3 | 4 |
| C. PSA test men with lower urinary tract symptoms? | 1 | 2 | 3 | 4 |
| D. PSA test men with a family history of prostate cancer? | 1 | 2 | 3 | 4 |
| E. PSA test men as part of an occupational health assessment? | 1 | 2 | 3 | 4 |
| F. PSA test men as a follow-up to medical procedures eg. *DRE or **TRUS? | 1 | 2 | 3 | 4 |

**C4: Do you perform any other blood tests routinely with a PSA test?** Yes 1 No 2

If Yes, which tests do you perform? Alkaline Phosphatase (PAP) 1 Creatine Kinase 2

Testerone 3 Other 4

If Other, please describe:

**C5: Do you usually use PSA test asymptomatic men for prostate cancer?**

Yes 1 No 2

If Yes, in what age groups? (Please tick as many as apply)

30-39 yrs 1 40-49 yrs 2 50-59 yrs 3 60-64 yrs 4

65-70 yrs 5 70-74 yrs 6 75-79 yrs 7 >80 yrs 8

*(For office use only)*

**C6. These questions relate to the consultation prior to PSA testing. Do you:**

|  | **Frequently** | **Sometimes** | **Rarely** | **Never** |
| --- | --- | --- | --- | --- |
| A. inform the patient that his PSA level is being checked, in a group of blood tests? | 1 | 2 | 3 | 4 |
| B. discuss the implication of the proposed PSA test, if it was to return as abnormal? | 1 | 2 | 3 | 4 |
| C. discuss the treatments of prostate cancer in general terms, at this stage? | 1 | 2 | 3 | 4 |
| D. ask, prior to testing, whether the patient has ejaculated in the preceding week?? | 1 | 2 | 3 | 4 |

* DRE=Digital Rectal Exam; ** TRUS = Transrectal ultrasound

**C7. For a man aged 55 at average risk of prostate cancer with a negative DRE, what is the lowest PSA level at which you would recommend a urological assessment?** *(Please tick one box only)*

| 2.5-3.9 ng/ml 1 |
| --- |
| 4.0-7.0 ng/ml 2 |
| 7.1-10.0 ng/ml 3 |
| >10.0 ng/ml 4 |
| Use laboratory reference range 5 |

**C8. For a man aged 65 at average risk of prostate cancer with a negative DRE, what is the lowest PSA level at which you would recommend a urological assessment?** *(Please tick one box only)*

| 2.5-3.9 ng/ml 1 |
| --- |
| 4.0-7.0 ng/ml 2 |
| 7.1-10.0 ng/ml 3 |
| >10.0 ng/ml 4 |
| Use laboratory reference range 5 |

**C9. Men who might have prostate cancer may present to their general practitioners in different ways. For the following presentations of men, please indicate which tests, if any you would be likely to perform:** *(Please tick as many boxes as apply)*

1. Mr Smith (aged 55 yrs) is fit and well and presents PSA 1

to you for his annual ‘checkup’. He has no significant DRE 2

medical or family history. Refer to a urologist 3

None of the above 4

Other 5

If Other, please state

1. Mr Jones (aged 55 yrs) is well but is concerned that he PSA 1

is at risk of getting cancer. His brother was diagnosed DRE 2

with prostate cancer this week and his aunt dies in her Refer to a urologist 3

forties of breast cancer. None of the above 4

Other 5

If Other, please state

1. Mr Jacobs (aged 55 yrs) has returned to your surgery PSA 1

for a follow-up after having radiotherapy for prostate DRE 2

cancer one year ago Refer to a urologist 3

None of the above 4

Other 5

If Other, please state

1. Mr Green (aged 55 yrs) is well and has reluctantly PSA 1

arrived to see you at your surgery. His wife has DRE 2

persuaded him to attend after she saw a documentary Refer to a urologist 3

on TV about prostate cancer. He has come to ask your None of the above 4

your advice about whether he should have a test done. Other 5

If Other, please state

**C10. To which laboratory do you primarily send your PSA test for analysis?**

| Letterkenny General |  1 | Adelaide and Meath |  14 |
| --- | --- | --- | --- |
| Sligo General |  2 | BSH Glasnevin |  15 |
| Cavan General |  3 | Mater Private |  16 |
| Mayo General |  4 | Mater Public |  17 |
| UCHG |  5 | St Vincent’s Public |  18 |
| BSH Galway |  6 | Our Lady of Lourdes |  19 |
| Claymon Laboratories |  7 | St James |  20 |
| Limerick Regional |  8 | Blackrock clinic |  21 |
| Kerry General |  9 | St Lukes, Rathgar |  22 |
| BSH, Tralee |  10 | St Lukes, Kilkenny |  23 |
| CUH |  11 | Beaumont | 24 |
| BSH, Cork |  12 | Waterford Regional |  25 |
| MUH, Cork |  13 | Other |  26 |

If Other, please state:

**C11. To which hospital do you primarily send men for prostatic investigation?**

| Letterkenny General |  1 | Adelaide and Meath |  14 |
| --- | --- | --- | --- |
| Sligo General |  2 | BSH Glasnevin |  15 |
| Cavan General |  3 | Mater Private |  16 |
| Mayo General |  4 | Mater Public |  17 |
| UCHG |  5 | St Vincent’s Public |  18 |
| BSH Galway |  6 | Our Lady of Lourdes |  19 |
| Claymon Laboratories |  7 | St James |  20 |
| Limerick Regional |  8 | Blackrock clinic |  21 |
| Kerry General |  9 | St Lukes, Rathgar |  22 |
| BSH, Tralee |  10 | St Lukes, Kilkenny |  23 |
| CUH |  11 | Beaumont | 24 |
| BSH, Cork |  12 | Waterford Regional |  25 |
| MUH, Cork |  13 | Other |  26 |

If Other, please state:

**C12. How often do you tink a PSA test should be performed in men ages 50 years and over?** *(Please tick one box).*

Annually or less 1

Every two years 2

More than every two years 3

When a man with risk factors develops symptoms 4

**C13.A. Do you, or your practice, have a policy on PSA testing?** Yes 1 No 2

B. If Yes, is it? A. a personal policy 1 a practice policy 2

B. an informal policy 1 a written policy 2

C. What year was it implemented*?*

Comments:

**C14.Has your own practice with regard to PSA testing changed in recent years?**

Yes 1 No 2

If Yes, please describe:

**C15.Is your PSA data computerised?** Yes 1 No 2

If Yes, since when?

**SECTION D: MANAGEMENT OF PSA RESULTS**

**M1. How do you respond to an abnormal PSA result?**

| A. Repeat the test | Yes 1 | No 2 |
| --- | --- | --- |
| B. Seek advices from the lab | Yes 1 | No 2 |
| C. Seek advice from urology | Yes 1 | No 2 |
| D. Counsel the patient and refer to urology | Yes 1 | No 2 |
| E. Refer directlty to urology outpatients | Yes 1 | No 2 |
| F. Other | Yes 1 | No 2 |

If Other, please comment:

**M2. How would you respond to the following scenarios**

1. A fit and healthy 57 year old man presents toyour Repeat PSA 1

surgery for the first time, with the result of some DRE 2

blood tests performed in his annual check up, 1 week Refer to a urologist 3

ago by his former GP. His PSA is 6ng/ml, free PSA 28%. Other 5

What would your preferred option be? *(Tick one box only)* If Other, please state

1. Which of the following treatments do you believe is the Watchful waiting 1

Most appropriate for a 65 year old man with localized Prostatectomy 2

prostate cancer but no other significant co-morbidity? Radiotherapy 3

*(Tick one box only)*  Hormone therapy 4

Other 5

If Other, please state

1. Which of the following treatments do you believe Watchful waiting 1

offers the greatest survival for a 55 year old man Prostatectomy 2

with localized prostate cancer but no other significant Radiotherapy 3

co-morbidity? *(Tick one box only)*  Hormone therapy 4

Other 5

If Other, please state

**M3. Has your own practice with regard to referral for urological assessment changed over recent years?**

Yes 1 No 2

If Yes, please describe:

Urological problems 4 Cancer detection/screening 5 Other 6

**M4. Have you had an asymptomatic patient aged under 60 years who had prostate cancer picked up via a PSA test?**

Yes 1 No 2

If Yes, has this influenced your practice in this matter? Yes 1 No 2

If Yes, please comment:

Urological problems 4 Cancer detection/screening 5 Other 6

**M5. Would you consider having a PSA test done yourself in the future?**

Yes 1 No 2 Not applicable 3

If Yes, please comment:

Urological problems 4 Cancer detection/screening 5 Other 6

**M6. This question concerns completion of death certificates. How would you describe the cause of death of the following men on their death certificates?**

**Primary cause Secondary cause**

1. A 70 year old man with localized prostate cancer for the

last 5 years dies of a heart attack. What do you put on the 1 2

death certificate?

1. A 70 year old man with metastatic prostate cancer dies

of a heart attack. What do you put on the death 1 2

certificate?

1. A 70 year old man with localised prostate cancer for the

last 5 years dies in a car crash. What do you put on the 1 2

death certificate?

1. A 70 year old man with metastatic prostate cancer

dies in a car crash. What do you put on the death 1 2

certificate?

**M7. Do you believe there is a need for national guidelines in relation to prostate cancer screening?**

Yes 1 No 2

Comment:

Urological problems 4 Cancer detection/screening 5 Other 6

**M8. Do you believe there is a need for national guidelines in relation to the use of PSA testing in General Practice?**

Yes 1 No 2

Comment:

Urological problems 4 Cancer detection/screening 5 Other 6

**M9: Do you feel you need more information with regard to:** *(Please tick as many as apply)*

PSA testing 1 Prostate cancer risk factors 2 Prostate cancer detection/diagnosis 3

Prostate cancer treatment 4 Prostate cancer survival 5 Other 6

If Other, please state:

Urological problems 4 Cancer detection/screening 5 Other 6

**M10. If there is anything else you wish to add about PSA testing or prostate cance, please use the box below.**

**Thank you very much for taking the time to participate in this survey.**

Please return your completed survey in the enclosed prepaid envelope to

Dr. Frances Drummond, Phd

Study coordinator,

National cancer Registry,

Elm Court, Boreenmanna road

Cork.

Tel: (021) 4703931; Fax: 021 4318016; email: [f.drummond@ncri.ie](mailto:f.drummond@ncri.ie); www.ncri.ie
